# Fall-Related Physical Function, Executive Function, and Myokine Responses in Older Women with Obesity Participating in a 16-Week Combined Aquatic Exercise Program: A Prospective Observational Study

**DOI:** 10.3390/medicina62091667

**Published:** 2026-08-31

**Authors:** Young-Min Seo, Wi-Young So, Kyeong-Lae Kim, Youn-Hwa Lee

**Affiliations:** 1 Department of Physical Education, College of Fourth, Korea National University of Education, Cheongju 28173, Republic of Korea; ymseo0113@gmail.com; 2 Department of Sports Medicine, College of Humanities, Korea National University of Transportation, Chungju 27469, Republic of Korea; wowso@ut.ac.kr

**Keywords:** combined aquatic exercise, fall-related physical function, executive function, healthy aging, irisin, myokines, obesity, older women

## Abstract

*Backgrand and Objective*: Aquatic exercise may be an effective strategy for promoting healthy aging among older women with obesity because it reduces joint loading while improving physical and functional capacity. In this prospective observational study, we examined longitudinal changes in fall-related physical function, executive function, and circulating myokine levels among older women with obesity according to their participation in a 16-week combined aquatic exercise program. *Materials and Methods*: Thirty older women with obesity were included in this prospective observational study and classified into an aquatic exercise group (*n* = 15) or a comparison group (*n* = 15) according to their participation in an existing community-based aquatic exercise program. Group membership was not assigned by the researchers. Participants in the aquatic exercise group attended the 16-week program, whereas those in the comparison group maintained their usual daily activities. Fall-related physical function, executive function, and circulating levels of the myokines irisin and interleukin-6 (IL-6) were assessed at baseline and at Weeks 8 and 16. Descriptive statistics were used to summarize participant characteristics, and two-way repeated-measures analyses of variance were performed to examine group differences, changes over time, and group × time interactions. When significant group × time interactions were observed, planned contrast analyses were conducted to examine changes across measurement time points. Statistical significance was set at *p* < 0.05. *Results*: Compared with the comparison group, the aquatic exercise group exhibited greater longitudinal improvements in several measures of fall-related physical function, including body composition, muscular function, and balance. However, no significant group × time interactions were observed for gait speed or endurance. Executive function demonstrated a significant group × time interaction (*p* < 0.001). Among the assessed myokines, irisin demonstrated a significant group × time interaction (*p* = 0.002), whereas IL-6 did not (*p* = 0.372), although IL-6 levels tended to decrease in the aquatic exercise group. *Conclusions*: Participation in the 16-week combined aquatic exercise program was associated with favorable longitudinal changes in several measures of fall-related physical function, executive function, and circulating irisin levels among older women with obesity. No significant group × time interactions were observed for gait performance or IL-6 levels. These findings suggest that participation in a community-based combined aquatic exercise program may be associated with favorable changes in selected indicators of healthy aging among older women with obesity.

## 1. Introduction

Interest in healthy aging is increasing rapidly as the global population continues to age. Notably, South Korea became a super-aged society in 2025, reflecting a substantial increase in its older adult population [1]. In parallel, obesity remains a major public health concern in the country, with the prevalence of obesity among adults reaching 38.4% in 2023 [2]. Research suggests that older women are particularly susceptible to the coexistence of obesity and reduced physical function because of hormonal changes associated with aging and menopause, reduced physical activity, and the progression of sarcopenia [3]. These conditions are closely associated with an increased risk of falls, loss of functional independence, and reduced quality of life [4], underscoring the need for effective strategies to preserve physical function and promote healthy aging in this population.

Age-related changes in body composition, particularly a decline in muscle mass and an increase in body fat, increase the risk of sarcopenic obesity [5]. In older adults, obesity is associated with an increased risk of cardiovascular disease and cognitive decline [6]. The loss of muscle mass in the context of obesity may further exacerbate metabolic risk factors and contribute to metabolic dysfunction [7]. Furthermore, obesity has been identified as an important contributor to increased fall risk, particularly among older women [6]. Reduced muscle strength and impaired balance can limit the ability to perform activities of daily living and increase susceptibility to falls. These impairments may perpetuate a vicious cycle of reduced physical activity and further functional decline [3]. Therefore, exercise-based strategies may play an important role in maintaining physical function and reducing fall risk among older women with obesity [8].

Aging is closely associated not only with declines in physical function but also with cognitive decline [9]. The frontal lobe, which plays a critical role in executive function, selective attention, and decision-making, is among the brain regions most susceptible to age-related atrophy [9]. Declines in executive function associated with frontal lobe atrophy may impair the ability to respond appropriately to changes in the environment, thereby increasing fall risk [10]. In contrast, regular physical activity has been associated with beneficial effects on frontal lobe function and cognitive performance [11,12]. Therefore, exercise-based strategies that address both physical and cognitive function may play an important role in promoting healthy aging [13].

Recently, exercise-induced myokines have received increasing attention because of their potential role in mediating the beneficial physiological effects of physical activity in older adults and promoting healthy aging [14]. Myokines are signaling molecules produced and secreted by skeletal muscle, particularly in response to muscle contraction, and are involved in the regulation of metabolic homeostasis and inflammatory responses [8]. Among these myokines, irisin plays an important role in energy metabolism and adipose tissue regulation; however, circulating irisin levels tend to decrease with aging and obesity [15,16]. Interleukin-6 (IL-6), by contrast, is a pleiotropic cytokine whose biological effects depend on the physiological context. Chronically elevated circulating IL-6 levels are associated with low-grade systemic inflammation and have been observed in older adults and individuals with low levels of physical activity [17]. Elevated resting IL-6 levels may adversely affect skeletal muscle mass and function [18]. Therefore, favorable modulation of myokine profiles through exercise may have important implications for obesity management and healthy aging in older adults [19].

Among various exercise modalities, aquatic exercise is considered particularly suitable for older adults with obesity because water buoyancy reduces joint loading while providing a supportive environment for improving physical function [20]. In addition, the buoyancy and resistance properties of water may facilitate balance training and enhance confidence in movement among older adults [21]. Previous studies have reported favorable effects of aquatic exercise on body composition, gait performance, immune function, and cognitive function in older adults [22,23]. Furthermore, meta-analytic evidence suggests that exercise training may favorably modulate circulating myokine levels, including irisin [24]. Notably, combined exercise programs incorporating both aerobic and resistance training may be particularly beneficial for maintaining physical function and managing body weight [25]. Moreover, moderate- to high-intensity exercise has been reported to improve body composition and functional fitness [26].

Despite these potential benefits, relatively few studies have examined longitudinal changes in fall-related physical function, executive function, and circulating myokine levels among older women with obesity participating in moderate- to high-intensity combined aquatic exercise programs in Korea. In particular, evidence regarding concurrent longitudinal changes in physical function, cognitive function, and physiological biomarkers remains limited [8]. Although previous studies have reported favorable outcomes associated with aquatic exercise in older adults, relatively few have comprehensively evaluated these multidimensional outcomes within the same observational follow-up period, particularly among older women with obesity. Therefore, this prospective observational study examined longitudinal changes in fall-related physical function, executive function, and circulating myokine levels among older women with obesity according to their participation in a 16-week community-based combined aquatic exercise program.

## 2. Materials and Methods

### 2.1. Design

This study employed a prospective observational, two-group longitudinal design to examine changes in fall-related physical function, executive function, and circulating myokine levels among older women with obesity according to their participation in a community-based combined aquatic exercise program. Participants were not assigned to the aquatic exercise or comparison group by the researchers; rather, group membership was determined by their participation or nonparticipation in the existing program. Assessments were conducted at baseline and at Weeks 8 and 16.

Participants in both groups were followed prospectively over the same 16-week period. Participants in the aquatic exercise group continued to attend their scheduled program, whereas those in the comparison group maintained their usual daily activities. The study procedures comprised participant recruitment and informed consent, baseline assessment, prospective follow-up, an interim assessment at Week 8, a final assessment at Week 16, and subsequent data analysis.

### 2.2. Participants

Before study initiation, ethical approval was obtained from the Institutional Review Board of Korea National University of Education, Cheongju, Republic of Korea (approval number: KNUE-202207-SBBR-0216-01; approval date: 11 July 2022). Participants were recruited from Goesan, Chungcheongbuk-do, Republic of Korea, through advertisements and informational notices distributed at local community facilities. Before enrollment, all participants received a detailed explanation of the study objectives, procedures, assessments, and potential risks and voluntarily provided written informed consent to participate.

Eligible individuals were women aged 65–75 years with a body fat percentage of ≥35% who had not engaged in regular structured physical activity during the previous 6 months, had no medical conditions that could limit participation in aquatic exercise or completion of the study assessments, and scored ≥24 on the Korean version of the Mini-Mental State Examination, indicating no apparent cognitive impairment. Body fat percentage was selected as the primary criterion for defining obesity because it more directly reflects adiposity than body mass index in older adults, in whom body composition may be affected by age-related reductions in skeletal muscle mass. Written informed consent was also obtained for the collection and use of biological specimens for research purposes.

An a priori sample size calculation was performed using G*Power software (version 3.1.9.7; Heinrich Heine University Düsseldorf, Düsseldorf, Germany). The calculation was based on a two-group repeated-measures analysis of variance (ANOVA) with three measurement time points, assuming a medium-to-large effect size (f = 0.32), a significance level (α) of 0.05, and a statistical power (1 − β) of 0.80. Under these assumptions, the minimum required sample size was estimated to be 22 participants [27]. The anticipated effect size was specified a priori for the expected group × time interaction across the repeated measurements. Because multiple physical, cognitive, and biochemical outcomes were evaluated, no single primary outcome was prospectively specified. To account for potential loss to follow-up, a total of 36 participants were enrolled.

Participants were classified according to their participation status in an existing community-based combined aquatic exercise program. Eighteen participants who elected to participate in the program were classified into the aquatic exercise group, whereas 18 eligible participants who did not participate in the program were classified into the comparison group. Participation in the aquatic exercise program was not assigned, randomized, or otherwise determined by the researchers. Accordingly, group membership reflected participants’ preexisting program participation status rather than researcher-directed group allocation. Both groups were followed prospectively over the same 16-week period and underwent assessments at the same time points.

**Figure 1:**
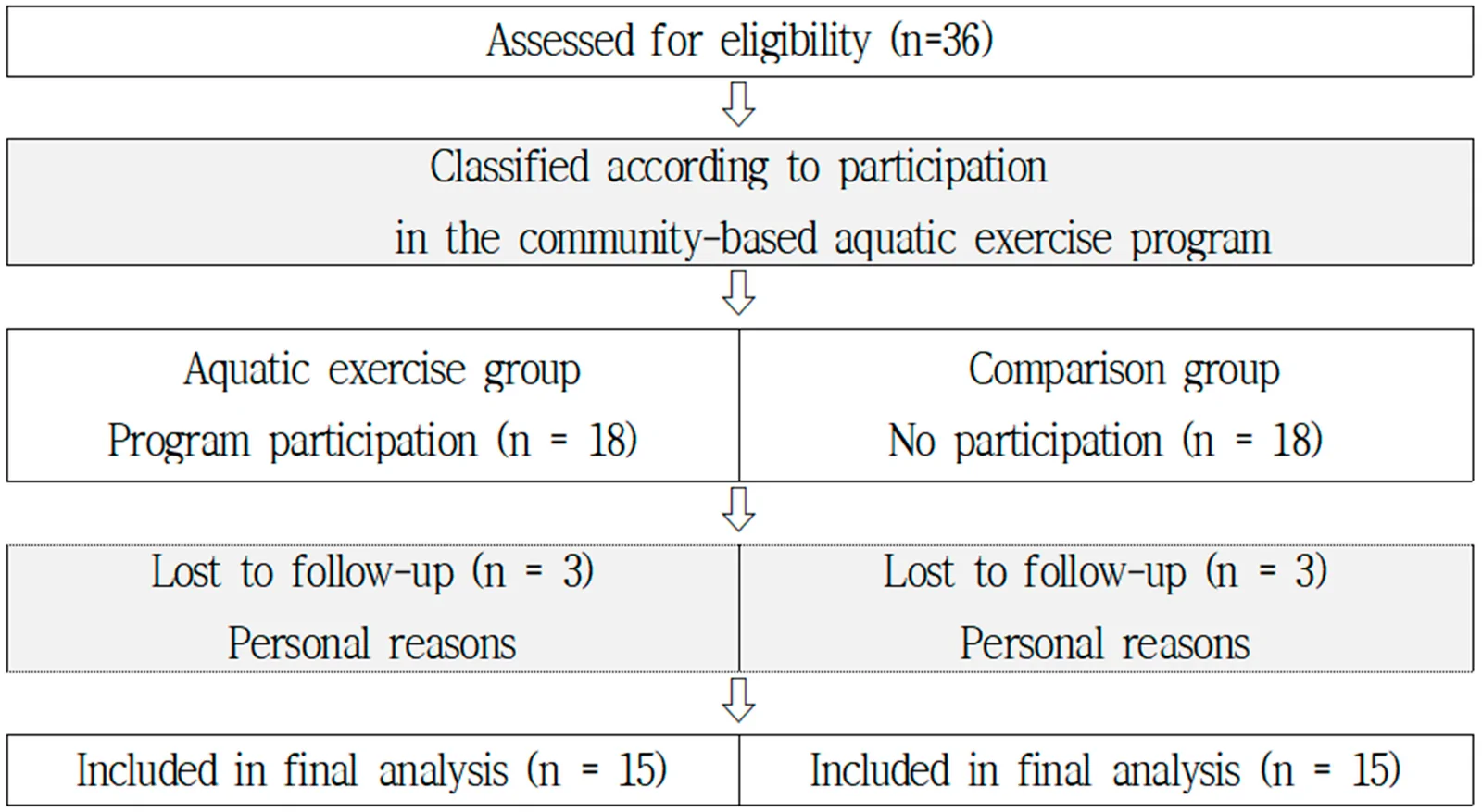
Participant flow diagram showing group classification based on participation in the aquatic exercise program, follow-up, and final analysis.

Because group membership was based on program participation and was therefore known to both participants and the researchers, neither participants nor outcome assessors were blinded to group status. Laboratory personnel conducting the biochemical analyses were blinded to group status, as described below. During the 16-week follow-up period, three participants in each group discontinued study participation for personal reasons unrelated to the aquatic exercise program or study assessments. Consequently, 30 participants—15 in the aquatic exercise group and 15 in the comparison group—completed all assessments and were included in the final analysis. Although the final sample size exceeded the minimum requirement estimated a priori, the actual statistical power may have differed from the anticipated power depending on the observed variance, within-participant correlations, and extent and pattern of missing data. The participant recruitment, group classification, follow-up, and analysis process is summarized in Figure 1.

### 2.3. Combined Aquatic Exercise Program

The community-based combined aquatic exercise program was conducted twice weekly for 16 weeks, with each session comprising a 5-min warm-up, a 50-min main exercise phase, and a 5-min cool-down. All exercise sessions were conducted in an indoor swimming pool with a water depth of 1.2 m and a water temperature of approximately 26 °C. The water level was maintained at approximately chest level for all participants.

No aquatic exercise equipment (e.g., kickboards, dumbbells, resistance paddles, or flotation devices) was used during the program. All exercises were performed using body movements alone, with water resistance serving as the sole source of external resistance. During the main exercise phase, participants were asked to report their perceived exertion once per session using the rating of perceived exertion (RPE) scale [28]. Based on the reported RPE, the instructor provided verbal feedback and adjusted movement amplitude, jump height, or movement speed as necessary to help participants maintain the target RPE range of 12–15. Individual RPE values were monitored during each session to help participants remain within the prescribed intensity range; however, these values were not formally recorded or retained as study data for subsequent analysis. During the cool-down phase, participants performed 5 min of low-intensity static stretching at an RPE of 10–11 to facilitate post-exercise recovery. Music tempo was adjusted according to the exercise phase, with a tempo of approximately 110 beats per minute (bpm) during the warm-up and cool-down phases and 120–135 bpm during the main exercise phase.

The warm-up phase primarily consisted of dynamic stretching exercises to facilitate physiological adaptation to the aquatic environment, whereas the cool-down phase consisted of low-intensity static stretching exercises to facilitate post-exercise recovery [29,30]. The main exercise phase incorporated aquarobics- and aquafit-based movements to provide balanced training of the upper and lower extremities. Exercise intensity and movement complexity were progressively increased throughout the 16-week program by modifying movement amplitude and speed, jump height, and the complexity of coordinated upper- and lower-extremity movements while maintaining the prescribed target intensity (RPE 12–15). Progression was individualized according to each participant’s exercise performance and perceived exertion. As participants adapted to the program, larger movement amplitudes, faster movement speeds, higher jumps (when appropriate), and more complex movement combinations were gradually introduced to increase hydrodynamic resistance without the use of external equipment.

All participants in the aquatic exercise group attended the sessions together, with approximately 15 participants per session. All exercise sessions were supervised by a certified exercise specialist, with an instructor-to-participant ratio of approximately 1:15, to ensure participant safety and adherence to the prescribed exercise intensity. All participants included in the final analysis completed the 16-week follow-up. Participants in the aquatic exercise group attended an average of 31.1 ± 1.0 of the 32 scheduled program sessions, corresponding to a mean attendance rate of 97.1 ± 3.2% (range: 29–32 sessions; 90.6–100%). The minimum attendance criterion (≥90% of scheduled program sessions) was predefined before study initiation. Attendance was monitored using records maintained by the research staff. Participants were monitored for adverse events and exercise-related discomfort throughout their participation in the program, and no exercise-related adverse events were reported during the 16-week follow-up period. The detailed components of the combined aquatic exercise program are presented in Table 1.

### 2.4. Outcome Measurements

#### 2.4.1. Fall-Related Physical Function Variables

Fall-related physical function was evaluated using measures of body composition, functional fitness, balance, and gait performance. Balance was further evaluated using the Berg Balance Scale (BBS), a validated instrument for assessing balance performance and fall risk. Body composition and Senior Fitness Test (SFT) assessments were conducted at the National Fitness Award Center under the supervision of the researchers. Gait and balance assessments were performed under standardized conditions by the same evaluator in the presence of licensed medical personnel to minimize measurement variability.

##### Body Composition

Body composition, particularly body weight and body fat percentage, was assessed to characterize obesity-related parameters. Measurements were obtained using a bioelectrical impedance analysis device (InBody 720, Biospace Co., Ltd., Seoul, Republic of Korea). Height (cm), body weight (kg), and body fat percentage (%) were measured according to the manufacturer’s instructions. Participants wore light clothing, removed all metallic accessories, and underwent the measurements in a fasting state before completing any other assessments.

##### Functional Fitness

Functional fitness was assessed using the SFT, which evaluates the physical capacities required to perform activities of daily living in older adults. Before testing, blood pressure was measured to ensure participant safety. Participants with a systolic blood pressure of ≥160 mmHg or a diastolic blood pressure of ≥100 mmHg were excluded from testing. In addition, participants completed the Physical Activity Readiness Questionnaire to identify potential medical risks associated with exercise participation.

Upper-extremity muscle function was evaluated using a handgrip strength test. Participants performed the test while standing with the shoulder adducted and the elbow fully extended. Maximal grip strength was measured twice for each hand in alternating order, and the highest value was recorded to the nearest 0.1 kg. Relative handgrip strength was calculated by dividing maximal grip strength by body weight and multiplying the resulting value by 100.

Lower-extremity muscle function was assessed using the 30-s chair stand test. Participants sat in the middle of an armless chair with their arms crossed over their chest. They were instructed to stand up fully and sit down repeatedly as many times as possible within 30 s. The total number of correctly completed repetitions was recorded.

Cardiorespiratory endurance was evaluated using the 2-min step test. Participants were instructed to raise their knees to a predetermined height corresponding to the midpoint between the patella and the iliac crest. The total number of correctly completed steps within 2 min was recorded.

Coordination was assessed using a figure-of-8 walk test. Participants were instructed to walk around cones positioned within a rectangular course and return to a seated position after completing the prescribed walking pattern. The total completion time was recorded to the nearest 0.001 s.

##### Balance

Balance was assessed using the BBS. The instrument consists of 14 performance-based tasks, each scored on a five-point ordinal scale ranging from 0 to 4. The total score ranges from 0 to 56, with higher scores indicating better balance performance. The assessment required approximately 15–20 min to complete [31].

##### Gait Performance

Gait performance was evaluated under standardized conditions after participants had achieved a resting heart rate, as monitored using a smartwatch. Participants were instructed to walk along a straight 10-m walkway that was 1 m wide. Gait speed was assessed by measuring the time required to walk 10 m, with completion time recorded in seconds. Gait endurance was assessed using the 6-min walk test. Participants walked repeatedly along the same 10-m course for 6 min, and the total distance covered was recorded in meters.

#### 2.4.2. Frontal Lobe Executive Function

Executive function was assessed using the Frontal-Executive Function Neuropsychological Test-II (FENT-II) [32]. The FENT-II is a standardized neuropsychological assessment battery designed to evaluate executive function based on the central executive model of cognitive processing. It consists of five subtests: the Stroop Test, Word Fluency Test, Design Fluency Test, Auditory Verbal Learning Test, and Complex Figure Test. Raw scores for each subtest were converted according to standardized procedures, and the Executive Function Quotient (EFQ) was calculated as the sum of the converted scores. Higher EFQ scores indicate better executive function. The FENT-II normative data are based on a large sample derived from the Korean population census, and the test has demonstrated acceptable reliability and criterion validity [32]. The same test battery was administered at each assessment time point. However, different initial consonants were used for the Word Fluency Test at each assessment to minimize potential learning or test-familiarization effects. The remaining subtests were administered using the same standardized procedures because alternative versions were not available within the FENT-II battery. Therefore, although efforts were made to minimize repeated-testing effects, potential practice effects associated with repeated cognitive assessment cannot be completely excluded.

##### Stroop Test

The Stroop Test was used to assess inhibitory control. Participants were required to selectively attend to one stimulus dimension while suppressing interference from a competing stimulus dimension. Performance was evaluated based on completion time and the number of errors. Shorter completion times and fewer errors indicated better inhibitory control.

##### Word Fluency Test

The Word Fluency Test was used to assess verbal cognitive flexibility. Participants were sequentially presented with three initial consonants, which differed across assessment time points to minimize potential learning or test-familiarization effects. They were asked to generate as many words as possible beginning with each consonant within 1 min. Repeated or incorrect responses were excluded from scoring. A greater number of correct responses indicated better performance [32].

##### Design Fluency Test

The Design Fluency Test was used to assess visuospatial cognitive flexibility and nonverbal generativity [32]. Participants were presented with test sheets containing five-dot patterns arranged within square frames. They were asked to generate as many unique designs as possible by connecting two or more dots while avoiding repeated designs. The test comprised three trials, with a time limit of 1 min per trial. The total number of correct, nonrepetitive designs was recorded, with a greater number indicating better performance.

##### Auditory Verbal Learning Test

The Auditory Verbal Learning Test was used to assess verbal learning and memory, including working memory processes [32]. Participants listened to a list of 15 nouns and were instructed to recall as many words as possible. The assessment comprised five learning trials followed by delayed recall and delayed recognition tasks. For the delayed recall task, participants were asked to recall the previously presented words after a 20-min delay without having been informed in advance of the subsequent memory test. The delayed recognition task was then administered using a recognition sheet containing target and distractor words. A greater number of correctly recalled and recognized words indicated better memory performance.

##### Complex Figure Test

The Complex Figure Test was used to assess visuoconstructive ability and executive aspects of planning and organization. Participants were instructed to copy a complex geometric figure presented on a stimulus card onto a blank sheet of paper. The stimulus figure was presented in a fixed orientation, and participants were not permitted to rotate it during the assessment. No time limit was imposed. Performance was evaluated based on planning and copy accuracy scores, with higher scores indicating better task performance [32].

#### 2.4.3. Myokines

For the analysis of circulating myokines, specifically irisin and IL-6, written informed consent for blood collection was obtained from all participants before study initiation. Baseline blood samples were collected in the morning 1 day before the start of the aquatic exercise program. Follow-up blood samples were collected at the corresponding Week 8 and Week 16 assessment time points in both groups. For participants in the aquatic exercise group, blood samples were collected 2 days after the most recent scheduled exercise session. Blood samples were collected under standardized conditions at all three time points. Participants were instructed to fast overnight for at least 8 h before blood collection and to refrain from vigorous physical activity for at least 24 h before sampling to minimize potential influence on circulating biomarker concentrations.

Before blood collection, participants were asked to rest quietly for at least 15 min. Subsequently, qualified medical personnel collected 10 mL of venous blood from an antecubital vein into serum separator tubes without anticoagulant. The blood was allowed to clot at room temperature for approximately 10 min and was then centrifuged at 3000 rpm for 15 min. The separated serum was aliquoted into microtubes and stored at −80 °C until analysis. All biochemical analyses were conducted at a certified medical laboratory.

Serum irisin concentrations were measured using a Human Irisin ELISA Kit (Phoenix Pharmaceuticals, Burlingame, CA, USA), and serum IL-6 concentrations were measured using a Human IL-6 ELISA Kit (Assaypro, St. Charles, MO, USA), according to the manufacturers’ instructions. Serum samples were analyzed in singlicate according to the manufacturers’ protocols. All serum samples obtained from the same participant across the three assessment time points were analyzed on the same assay plate within a single analytical batch to minimize inter-assay variability. The analytical ranges of the assays were 0.1–1000 ng/mL for irisin and 3.906–250 pg/mL for IL-6. Before analysis, all reagents were equilibrated to room temperature (20–25 °C), and absorbance was measured at 450 nm using a Model 680 Microplate Reader (Bio-Rad Laboratories, Hercules, CA, USA). To minimize detection bias, laboratory personnel performing the biochemical analyses were blinded to group status, and all samples were labeled using coded participant identification numbers.

### 2.5. Statistical Analysis

Statistical analyses were performed using IBM SPSS Statistics for Windows, version 31.0 (IBM Corp., Armonk, NY, USA), using a complete-case approach. Only participants who completed all scheduled assessments during the 16-week follow-up period were included in the final analyses. Participants with incomplete follow-up data were excluded because outcome data after discontinuation were unavailable. Accordingly, sensitivity analyses addressing the potential impact of missing follow-up data could not be performed retrospectively. Descriptive data are presented as means and standard deviations. Independent-samples *t*-tests were used to compare baseline characteristics between the aquatic exercise and comparison groups. To examine longitudinal differences between the groups, a two-way repeated-measures analysis of variance (ANOVA) was performed to evaluate the main effects of group and time and the group × time interaction. Normality was assessed using the Shapiro–Wilk test, homogeneity of variance was evaluated using Levene’s test, and sphericity was assessed using Mauchly’s test. When the assumption of sphericity was violated, the Greenhouse–Geisser correction was applied. Degrees of freedom (df), partial eta squared (ηp^2^), and corresponding *p*-values are reported for all ANOVA results. When significant group × time interactions were observed, planned contrast analyses were performed to compare changes across measurement time points. Statistical significance was set at *p* < 0.05.

## 3. Results

### 3.1. Participant Characteristics, Study Variables, and Baseline Comparability Between the Two Groups

A total of 30 participants (15 per group) completed the 16-week follow-up and were included in the final analysis. Among participants in the aquatic exercise group, attendance at the community-based aquatic exercise program was high, with a mean attendance rate of 97.1 ± 3.2% (range: 90.6–100%). No exercise-related adverse events were reported among program participants during the 16-week follow-up period. Baseline participant characteristics and study variables are presented in Table 2. No statistically significant between-group differences were observed in the assessed baseline characteristics or study variables (*p* > 0.05).

### 3.2. Changes in Fall-Related Physical Function Variables

Changes in fall-related physical function variables in the aquatic exercise and comparison groups are presented in Table 3. Significant group × time interactions were observed for body weight (*p* = 0.004), body fat percentage (*p* < 0.001), upper-extremity muscle function (*p* = 0.049), lower-extremity muscle function (*p* = 0.009), cardiorespiratory endurance (*p* = 0.037), coordination (*p* = 0.001), and balance (*p* = 0.001). In contrast, no significant group × time interactions were observed for gait completion time (*p* = 0.761) or gait endurance (*p* = 0.188).

Regarding body composition, body weight and body fat percentage decreased significantly over time in the aquatic exercise group. In the comparison group, body fat percentage increased significantly from baseline to Week 16, whereas body weight showed a nonsignificant increasing trend. Planned contrast analyses revealed significant reductions in body weight from baseline to Week 8 (*p* = 0.013) and from baseline to Week 16 (*p* < 0.001) in the aquatic exercise group. Similarly, body fat percentage decreased significantly from baseline to Week 8 (*p* = 0.009) and from baseline to Week 16 (*p* < 0.001) in the aquatic exercise group.

Regarding functional fitness, significant improvements from baseline to Week 16 were observed in upper-extremity muscle function (*p* = 0.042), lower-extremity muscle function (*p* = 0.007), and cardiorespiratory endurance (*p* = 0.006) in the aquatic exercise group. In contrast, coordination completion time increased significantly from baseline to Week 16 in the comparison group (*p* = 0.001), indicating poorer coordination performance.

Balance improved significantly from baseline to Week 8 (*p* < 0.001), from Week 8 to Week 16 (*p* = 0.001), and from baseline to Week 16 (*p* < 0.001) in the aquatic exercise group, indicating progressive improvement throughout the 16-week follow-up period.

### 3.3. Changes in Executive Function

Table 4 presents the EFQ results for both groups across the 16-week follow-up period. A significant group × time interaction (*p* < 0.001) and a significant main effect of time (*p* < 0.001) were observed, whereas the main effect of group was not significant (*p* = 0.120). Planned contrast analyses indicated significant increases in EFQ across all assessment intervals in the aquatic exercise group: from baseline to Week 8 (*p* < 0.001), from Week 8 to Week 16 (*p* = 0.012), and from baseline to Week 16 (*p* < 0.001). In the comparison group, a significant increase was observed only from baseline to Week 16 (*p* = 0.005). EFQ increased from 96.13 ± 7.07 at baseline to 124.93 ± 15.02 at Week 16 in the aquatic exercise group and from 100.06 ± 10.01 at baseline to 111.60 ± 12.56 at Week 16 in the comparison group, indicating a greater longitudinal increase in EFQ in the aquatic exercise group.

### 3.4. Changes in Myokines

Changes in circulating myokine concentrations in the aquatic exercise and comparison groups are presented in Table 5. A significant group × time interaction was observed for irisin (*p* = 0.002), whereas neither the main effect of group (*p* = 0.462) nor the main effect of time (*p* = 0.160) was statistically significant. In contrast, no significant main effect of group (*p* = 0.286), main effect of time (*p* = 0.125; Greenhouse–Geisser corrected), or group × time interaction (*p* = 0.372) was observed for IL-6. Planned contrast analyses revealed significant increases in irisin concentrations from Week 8 to Week 16 (*p* = 0.005) and from baseline to Week 16 (*p* = 0.002) in the aquatic exercise group. In the comparison group, irisin concentrations decreased significantly from baseline to Week 8 (*p* = 0.046) and from baseline to Week 16 (*p* = 0.036). In the aquatic exercise group, irisin concentrations increased from 4.15 ± 0.57 ng/mL at baseline to 4.99 ± 0.91 ng/mL at Week 16, whereas concentrations in the comparison group decreased from 4.75 ± 0.97 ng/mL at baseline to 4.12 ± 0.50 ng/mL at Week 16. For IL-6, no statistically significant main effects or group × time interaction were observed, although concentrations decreased numerically in the aquatic exercise group over the 16-week follow-up period.

## 4. Discussion

### 4.1. Changes in Fall-Related Physical Function Variables

This study examined longitudinal changes in fall-related physical function variables among older women with obesity according to their participation in a 16-week aquatic combined exercise program. The fall-related physical function variables included body composition, functional fitness, balance, and gait performance. These variables are closely interrelated and are widely considered important for maintaining physical function and reducing fall risk in older adults. Aging is generally accompanied by reductions in muscle mass and increases in body fat, and obesity may exacerbate the associated functional impairments, contributing to diminished functional fitness and an increased risk of falls [33].

In the present study, body weight and body fat percentage decreased significantly over time in the aquatic exercise group. In contrast, body fat percentage increased significantly from baseline to Week 16 in the comparison group, whereas body weight showed a nonsignificant increasing trend. These findings are consistent with those of previous studies reporting favorable changes in body weight and body fat percentage following aquatic exercise [34,35,36]. The aquatic environment facilitates exercise with reduced joint loading because of water buoyancy while providing resistance to movement, which may support energy expenditure during exercise. The reductions in body weight and body fat percentage observed in the aquatic exercise group suggest that participation in aquatic combined exercise may be associated with favorable changes in body composition among older women with obesity.

Balance is considered one of the most important physical factors associated with fall risk. In the present study, BBS scores improved significantly across all assessment intervals in the aquatic exercise group, whereas no significant changes were observed in the comparison group.

BBS scores are generally inversely associated with fall risk, with higher scores indicating better balance performance and a lower likelihood of falling [37]. Although the participants in this study were independently functioning older adults and had relatively high BBS scores at baseline, their scores showed progressive increases throughout the 16-week follow-up period. These findings suggest that participation in aquatic combined exercise may be associated with favorable changes in balance and fall-related physical function among older women with obesity. Notably, the aquatic exercise group demonstrated a mean increase of 4.66 points in BBS score from baseline to Week 16. Given that higher BBS scores reflect better balance performance and are associated with a lower risk of falls, the observed increase may represent a potentially meaningful change in functional balance.

Regarding functional fitness, significant improvements were observed in lower-extremity muscle function and cardiorespiratory endurance, whereas coordination performance also showed favorable changes. In contrast, although upper-extremity muscle function showed a significant group × time interaction, only the change from baseline to Week 16 reached statistical significance in the planned contrast analysis (*p* = 0.042). These findings may be partly related to the characteristics of aquatic exercise, which often involves repeated lower-extremity-dominant movements. A previous study reported significant improvements in knee extensor and flexor muscle function following a 12-week exercise intervention in middle-aged women with obesity and suggested that these adaptations were related to the repeated use of the lower-extremity musculature [38]. Similarly, the aquatic combined exercise program examined in the present study incorporated repeated lower-body activities, such as walking, jumping, and aquarobic movements, which may have contributed to the favorable longitudinal changes observed in lower-extremity muscle function. These findings are also consistent with previous studies of aquatic exercise that reported greater improvements in lower-extremity function than in upper-extremity function [39,40]. Furthermore, coordination performance improved in the aquatic exercise group, whereas it declined in the comparison group. Because coordination is important for performing daily activities that require the simultaneous control of upper- and lower-extremity movements, better coordination may contribute to safer mobility and greater functional independence in older adults. Collectively, these findings suggest that regular participation in exercise may be associated with the maintenance or improvement of functional fitness in older adults.

No significant changes were observed in gait completion time or gait endurance. These findings may be related to the characteristics of the study population. Although the participants were community-dwelling older women with obesity who were functionally independent and had no limitations precluding participation in moderate- to vigorous-intensity exercise, their baseline balance and gait performance were relatively high, suggesting a potential ceiling effect that may have limited the ability to detect further improvements in gait-related outcomes during the 16-week follow-up period. Nevertheless, gait endurance increased numerically in the aquatic exercise group despite this potential ceiling effect. However, whether participants with lower baseline gait performance would derive greater functional benefits from aquatic combined exercise remains to be determined. Previous research has demonstrated that walking performance in older adults is strongly associated with lower-extremity muscle strength and power, with better lower-extremity function being associated with greater mobility and walking capacity. Therefore, maintaining or improving lower-extremity muscle function, together with appropriate weight management, may play an important role in mitigating age-related functional decline [41].

Overall, the reductions in body weight and body fat percentage, along with the improvements in lower-extremity muscle function, cardiorespiratory endurance, coordination performance, and balance, suggest that participation in aquatic combined exercise was associated with favorable longitudinal changes in functional fitness and selected fall-related physical function variables among older women with obesity. In particular, participation in combined exercise performed in an aquatic environment was associated with favorable longitudinal changes in body composition and functional fitness among older women with obesity. From a clinical perspective, improvements in balance, lower-extremity muscle function, and cardiorespiratory endurance may contribute to maintaining functional independence and mobility, which are important components of healthy aging among community-dwelling older adults. Although statistical significance does not necessarily indicate clinical importance, the observed changes in these functional outcomes, together with the moderate-to-large effect sizes for several outcomes, suggest that the longitudinal differences may have potential clinical relevance.

### 4.2. Changes in Executive Function

The present study examined longitudinal changes in executive function among older women with obesity according to their participation in a 16-week aquatic combined exercise program. In the aquatic exercise group, executive function increased significantly from baseline to Week 8 and from Week 8 to Week 16. In contrast, executive function in the comparison group increased significantly only from baseline to Week 16. The increase observed in the comparison group may partially reflect practice effects associated with repeated administration of the cognitive assessments. Although practice effects cannot be completely excluded, the use of different initial consonants in the Word Fluency Test reduced repeated exposure to identical test stimuli. Therefore, although the greater longitudinal increase observed in the aquatic exercise group was associated with program participation, the observed differences between groups may reflect a combination of exercise-related adaptations, repeated-testing effects, and other unmeasured factors that differed between the groups. In addition, the potential influence of assessor expectancy and the lack of blinded outcome assessment should be considered when interpreting these findings.

Obesity is recognized as an important risk factor for cognitive decline. It is associated with an increased risk of cardiovascular and metabolic diseases, which may contribute to cerebrovascular dysfunction and, consequently, cognitive decline [35,36,37,38,39,40,41,42,43,44]. In older women, the decline in estrogen levels following menopause has been associated with adverse cardiovascular changes and cognitive decline [45]. Therefore, the reductions in body weight and body fat percentage, together with the improvement in cardiorespiratory endurance observed in the aquatic exercise group, may have been associated with the favorable longitudinal changes in executive function.

The favorable longitudinal changes in executive function observed in this study are generally consistent with findings from previous studies. Several studies have reported beneficial associations between physical activity or exercise and cognitive function in older adults. Exercise has been associated with improved cognitive performance and may represent a potentially useful strategy for maintaining cognitive health and attenuating age-related cognitive decline in later life [46,47]. Furthermore, previous studies have reported favorable changes in various domains of cognitive function following aquatic combined exercise in older adults, including executive function, memory, and overall cognitive performance [48,49,50].

The aquatic combined exercise program examined in this study incorporated aerobic and resistance exercises, as well as repetitive rhythmic movements performed to music. Such activities may simultaneously engage visual, auditory, and proprioceptive processing and increase the cognitive demands placed on prefrontal cortical networks during exercise. In addition, previous studies have suggested that rhythm-based exercise may support cognitive function and that greater movement complexity is associated with increased activation of prefrontal brain regions [51,52].

Overall, the findings of this study suggest that participation in a 16-week aquatic combined exercise program was associated with favorable longitudinal changes in executive function among older women with obesity. Notably, significant increases in executive function were observed from baseline to Week 8 and from Week 8 to Week 16. These findings suggest that participation in aquatic combined exercise may be associated with the maintenance or improvement of executive function among older women with obesity. Because executive function is closely associated with safe mobility, independent functioning in daily life, and the successful performance of complex tasks in later life, these changes may also have meaningful implications for healthy aging. Although clinically meaningful thresholds for the EFQ have not been established, the magnitude of the observed increase in the aquatic exercise group suggests potential functional relevance; however, its clinical significance cannot be established from the present findings alone.

### 4.3. Changes in Myokines

The present study examined longitudinal changes in circulating myokine concentrations among older women with obesity according to their participation in a 16-week aquatic combined exercise program. Circulating irisin concentrations increased significantly from baseline to Week 16 in the aquatic exercise group, whereas no statistically significant longitudinal changes were observed in IL-6 concentrations. Because blood samples were collected following an overnight fast and at least 2 days after the most recent scheduled exercise session in the aquatic exercise group, the measured concentrations were less likely to reflect acute exercise-induced fluctuations and may instead reflect longer-term physiological changes occurring during the follow-up period.

Irisin has attracted considerable attention as an exercise-responsive myokine involved in metabolic regulation. Previous studies have reported lower circulating irisin concentrations in individuals with obesity and older adults [16,53]. Irisin has been implicated in the regulation of energy metabolism through mechanisms that may include the browning of white adipose tissue and the regulation of glucose metabolism and has therefore been proposed as a potential marker of metabolic health [54]. In addition, circulating irisin concentrations have been associated with habitual physical activity and skeletal muscle function [55].

In the present study, circulating irisin concentrations increased significantly from Week 8 to Week 16 and from baseline to Week 16 in the aquatic exercise group. In contrast, circulating irisin concentrations decreased significantly from baseline to Week 8 and from baseline to Week 16 in the comparison group. These contrasting longitudinal patterns occurred alongside reductions in body weight and body fat percentage and improvements in functional fitness in the aquatic exercise group. Previous studies have reported that regular exercise may stimulate irisin secretion, with combined aerobic and resistance exercise potentially eliciting greater responses than either modality alone [56]. Studies of aquatic exercise interventions have also reported increases in circulating irisin concentrations following exercise, accompanied by improvements in physical function and neurotrophic responses [57]. Furthermore, increases in circulating irisin concentrations have been reported following combined exercise programs [58]. Collectively, this evidence is consistent with the findings of the present study and suggests that participation in aquatic combined exercise may be associated with favorable changes in circulating irisin and related metabolic processes among older women with obesity.

No statistically significant longitudinal changes were observed in IL-6 concentrations. Although IL-6 concentrations decreased numerically in the aquatic exercise group, the change was not statistically significant and should therefore be interpreted cautiously. Circulating IL-6 concentrations may increase transiently following acute exercise, whereas resting concentrations may decrease following longer-term exercise training [59]. Regular exercise has also been associated with favorable changes in chronic low-grade inflammation [60]. Therefore, the numerical decrease observed in the present study may be consistent with longer-term physiological adaptations to exercise; however, this interpretation remains speculative. Further studies with larger sample sizes are needed to determine whether aquatic combined exercise is associated with longitudinal changes in resting IL-6 concentrations among older women with obesity.

Several studies have reported no statistically significant changes in IL-6 concentrations following short-term exercise interventions. Vasić et al. [61] reported no significant changes in IL-6 concentrations following an aquatic exercise intervention. Similarly, Mahendra et al. [62] observed no significant reductions in IL-6 concentrations in older adults following an 8-week exercise program. Studies conducted in Korea have also reported similar findings; although IL-6 concentrations decreased following exercise interventions, these reductions were not statistically significant [63]. Likewise, Lee et al. [64] reported numerical decreases in inflammatory markers following a combined exercise intervention, but these changes were not statistically significant.

In contrast, studies employing longer-duration or higher-intensity exercise programs have reported significant reductions in IL-6 concentrations. Long-term resistance training has been associated with reductions in circulating inflammatory markers [19]. Significant decreases in IL-6 concentrations have also been reported following combined aerobic and resistance exercise interventions [65]. Therefore, the absence of statistically significant changes in IL-6 in the present study suggests that participation in the 16-week aquatic combined exercise program was not associated with detectable longitudinal changes in resting IL-6 concentrations under the conditions examined.

Overall, the findings of this study suggest that participation in a 16-week aquatic combined exercise program was associated with favorable longitudinal changes in circulating irisin concentrations among older women with obesity, which may reflect underlying physiological adaptations. However, although IL-6 concentrations decreased numerically in the aquatic exercise group, the present findings do not provide direct evidence of an anti-inflammatory effect. Despite these favorable findings, several limitations should be acknowledged.

First, because this was a prospective observational study in which participants were not randomly assigned to groups, self-selection bias and residual confounding cannot be excluded. Participants who elected to participate in the aquatic exercise program may have differed from nonparticipants in unmeasured characteristics, such as motivation, health behaviors, or lifestyle factors. Therefore, the observed group × time differences should be interpreted as associations rather than as evidence of causal effects of the aquatic exercise program. Second, the study included a relatively small sample recruited from a single geographic area, which may limit the generalizability of the findings. In addition, the participants were community-dwelling older women who were relatively healthy and able to participate independently in the exercise program. Therefore, the findings may not be generalizable to older men, frailer older adults, individuals with cognitive impairment, or those with multiple chronic conditions. Third, neither the outcome assessor nor the data analyst was blinded to group status, which may have introduced detection or analytical bias. Fourth, although participants in both groups were instructed to maintain their usual dietary and lifestyle habits and to refrain from initiating any new structured exercise programs throughout the 16-week follow-up period, adherence to these instructions was not objectively monitored. Therefore, the potential influence of unmeasured lifestyle-related confounders, including changes in physical activity, dietary intake, medication use, and participation in other health-related activities, cannot be excluded. Fifth, no long-term follow-up assessment was performed; therefore, the persistence of the observed changes beyond the 16-week follow-up period remains unknown. Sixth, statistical analyses were performed using a complete-case approach because follow-up outcome data were unavailable for participants who discontinued participation. Therefore, sensitivity analyses addressing missing follow-up data could not be performed retrospectively. Consequently, attrition and selection bias, as well as overestimation of the observed group differences, cannot be excluded, and the findings should be interpreted with appropriate caution. Seventh, because no formal adjustment for multiple comparisons was applied across the multiple outcomes evaluated in this study, an increased risk of Type I error cannot be excluded. Therefore, findings with borderline statistical significance, particularly those from secondary outcomes, should be interpreted cautiously.

## 5. Conclusions

This prospective observational study examined longitudinal changes in fall-related physical function variables, executive function, and circulating myokines among older women with obesity according to their participation in a 16-week community-based aquatic combined exercise program. Compared with the comparison group, the aquatic exercise group showed more favorable longitudinal changes in body fat percentage, lower-extremity muscle function, cardiorespiratory endurance, coordination, and balance ability, whereas no significant group × time interactions were observed for gait speed or gait endurance. Executive function and circulating irisin concentrations also showed more favorable longitudinal changes in the aquatic exercise group, whereas no significant group × time interaction was observed for IL-6. Overall, participation in the 16-week aquatic combined exercise program was associated with favorable longitudinal changes in selected fall-related physical function variables, executive function, and circulating irisin concentrations among relatively healthy older women with obesity. Given the observational design and the potential for self-selection bias and residual confounding, these findings should not be interpreted as evidence of causal effects of the aquatic exercise program. Further studies with larger and more diverse samples, longer follow-up periods, and study designs that more effectively address potential confounding are warranted to validate and extend these findings.

## Figures and Tables

**Table 1:** Overview of the 16-Week Combined Aquatic Exercise Program.

Phase	Exercise Type		Description	Intensity
Warm-up (5 min)	Warm-up exercise		Dynamic stretching	Rating of perceived exertion: 10–11
Main exercise (50 min)	Aerobic exercises (30 min)	Running	Various aquarobics-based movements were combined to provide moderate- to vigorous-intensity aerobic exercise.	Rating of perceived exertion: 12–15
		High knees		
		Jumping push-pull		
		Kicks (front, back, shuffle)		
		Swings (inward, outward)		
		Side steps		
		Heel–toe twists		
		Jumping jacks		
		Twist jumping jacks		
		Leg swings (front, back, left, right)		
		Leg curls (back, side)		
		Leg curl and push-up		
		Sprint		
	Muscular exercises (20 min)	Squat	Resistance exercises were modified to maximize resistance provided by the water.	
		Wide squat		
		Standing toe touch		
		Good morning		
		Standing crunch (front, side)		
		Big clap and bird clap		
		Arm circles		
		Standing skater		
		Standing sprinter		
		Elbow-to-knee twist		
		Pull-down and side tap		
Cool-down (5 min)	Cool-down exercise		Static stretching	Rating of perceived exertion: 10–11

**Table 2:** Participant Characteristics and Study Variables at Baseline.

Variable		Aquatic Exercise Group (*n* = 15)	Comparison Group (*n* = 15)	t	*p*
Age (years)		68.33 ± 2.82	67.60 ± 2.87	0.933	0.343
Anthropometric characteristics	Height (cm)	156.78 ± 3.87	155.09 ± 4.75	1.143	0.294
	Weight (kg)	67.26 ± 6.10	65.10 ± 5.44	1.019	0.317
Fall-related physical function variables	Body fat percentage (%)	40.99 ± 3.98	38.33 ± 3.65	1.907	0.067
	Upper-extremity muscle function (%)	38.68 ± 8.34	40.74 ± 5.82	−0.784	0.440
	Lower-extremity muscle function (repetitions)	15.66 ± 4.56	15.06 ± 2.86	0.431	0.670
	Cardiorespiratory endurance (repetitions)	105.93 ± 13.99	101.66 ± 7.73	1.034	0.313
	Coordination (s)	27.01 ± 3.10	26.54 ± 3.63	0.380	0.707
	Balance (score)	47.40 ± 1.50	46.60 ± 3.01	0.919	0.366
	Gait completion time (s)	9.03 ± 1.06	9.28 ± 1.01	−0.750	0.459
	Gait endurance (m)	339.25 ± 26.20	341.08 ± 22.92	−0.204	0.840
Executive function	Executive Function Quotient (score)	96.13 ± 7.07	100.07 ± 10.01	−1.242	0.225
Myokines	Irisin (ng/mL)	4.15 ± 0.57	4.75 ± 0.97	−1.927	0.071
	Interleukin-6 (pg/mL)	6.53 ± 3.30	6.98 ± 2.78	−0.380	0.708

Data are presented as means ± standard deviations. Baseline differences between the two groups were assessed using independent-samples *t*-tests.

**Table 3:** Longitudinal Changes in Fall-Related Physical Function Variables in the Aquatic Exercise and Comparison Groups.

Variables	Group	Baseline	Week 8	Week 16	Effect	df	F	*p*	ηp^2^	Planned Contrasts
Weight (kg)	EG (*n* = 15)	67.26 ± 6.10	62.65 ± 4.25	60.22 ± 5.78	Group (A)	1, 28	7.578	0.010 *	0.213	EG: 0–8 *, 0–16 ***
	CG (*n* = 15)	65.10 ± 5.44	66.95 ± 4.94	67.37 ± 5.81	Time (B)	2, 56	1.522	0.227	0.052	
					A × B	2, 56	6.024	0.004 **	0.177	
Body Fat Percentage (%)	EG (*n* = 15)	40.99 ± 3.98	37.76 ± 3.25	36.17 ± 2.10	Group (A)	1, 28	8.524	0.007 **	0.233	EG: 0–8 **, 0–16 *** CG: 0–16 *
	CG (*n* = 15)	38.33 ± 3.65	41.12 ± 4.20	41.69 ± 4.00	Time (B)	2, 56	0.298	0.743	0.011	
					A × B	2, 56	9.735	<0.001 ***	0.258	
Upper-Extremity Muscle Function (%)	EG (*n* = 15)	38.68 ± 8.34	41.08 ± 7.25	44.76 ± 8.17	Group (A)	1, 28	0.381	0.542	0.013	EG: 0–16 *
	CG (*n* = 15)	40.74 ± 5.82	41.14 ± 6.01	39.28 ± 5.79	Time (B)	2, 56	1.125	0.332	0.039	
					A × B	2, 56	3.186	0.049 *	0.102	
Lower-Extremity Muscle Function (repetitions)	EG (*n* = 15)	15.66 ± 4.56	19.20 ± 4.50	20.80 ± 5.74	Group (A)	1, 28	15.450	0.001 **	0.356	EG: 0–16 **
	CG (*n* = 15)	15.06 ± 2.86	14.33 ± 3.57	13.73 ± 4.43	Time (B)	2, 56	1.819	0.172	0.061	
					A × B	2, 56	5.069	0.009 **	0.153	
Cardiorespiratory Endurance (repetitions)	EG (*n* = 15)	105.93 ± 13.99	109.73 ± 12.98	118.80 ± 9.37	Group (A)	1, 28	15.110	0.001 **	0.350	EG: 0–16 **
	CG (*n* = 15)	101.66 ± 7.73	95.26 ± 13.78	103.46 ± 2.72	Time (B)	2, 56	7.990	0.001 **	0.222	
					A × B	2, 56	3.492	0.037 *	0.111	
Coordination (s)	EG (*n* = 15)	27.01 ± 3.10	24.57 ± 4.68	24.15 ± 2.38	Group (A)	1, 28	25.835	<0.001 ***	0.480	EG: 0–16 * CG: 8–16 *, 0–16 **
	CG (*n* = 15)	26.54 ± 3.63	28.29 ± 3.39	31.46 ± 4.18	Time (B)	2, 56	1.062	0.352	0.037	
					A × B	2, 56	7.839	0.001 **	0.219	
Balance (score)	EG (*n* = 15)	47.40 ± 1.50	49.86 ± 1.40	52.06 ± 2.25	Group (A)	1, 28	17.396	<0.001 ***	0.383	EG: 0–8 ***, 8–16 **, 0–16 ***
	CG (*n* = 15)	46.60 ± 3.01	47.00 ± 3.29	46.86 ± 3.15	Time (B)	1.64, 45.77	11.052	<0.001 ***	0.283	
					A × B	1.64, 45.77	8.726	0.001 **	0.238	
Gait Completion Time (s)	EG (*n* = 15)	9.03 ± 1.06	9.17 ± 1.28	9.07 ± 0.58	Group (A)	1, 28	0.224	0.639	0.008	—
	CG (*n* = 15)	9.28 ± 1.01	9.18 ± 1.39	9.27 ± 0.90	Time (B)	2, 56	0.011	0.989	<0.001	
					A × B	2, 56	0.275	0.761	0.010	
Gait Endurance (m)	EG (*n* = 15)	339.25 ± 26.20	340.22 ± 26.02	347.75 ± 32.12	Group (A)	1, 28	0.045	0.833	0.002	—
	CG (*n* = 15)	341.08 ± 22.92	340.93 ± 21.34	339.56 ± 25.09	Time (B)	1.28, 35.94	0.868	0.384	0.030	
					A × B	1.28, 35.94	1.796	0.188	0.060	

Values are presented as means ± standard deviations. EG = aquatic exercise group (participants in the community-based aquatic exercise program); CG = comparison group (participants who did not participate in the aquatic exercise program). Group classification was based on participants’ program participation status and was not assigned by the researchers. The main effects of group (A) and time (B) and the group × time interaction (A × B) were analyzed using a two-way repeated-measures analysis of variance. When the assumption of sphericity was violated, the Greenhouse–Geisser correction was applied. When significant group × time interactions were observed, planned contrast analyses were performed to compare changes across measurement time points (0–8 = baseline to Week 8; 8–16 = Week 8 to Week 16; 0–16 = baseline to Week 16). * *p* < 0.05, ** *p* < 0.01, *** *p* < 0.001.

**Table 4:** Longitudinal Changes in Executive Function in the Aquatic Exercise and Comparison Groups.

Variable	Group	Baseline	Week 8	Week 16	Effect	df	F	*p*	ηp^2^	Planned Contrasts
Executive Function Quotient (score)	EG (*n* = 15)	96.13 ± 7.07	113.66 ± 11.89	124.93 ± 15.02	Group (A)	1, 28	2.575	0.120	0.084	EG: 0–8 ***, 8–16 **, 0–16 *** CG: 0–16 **
	CG (*n* = 15)	100.06 ± 10.01	105.66 ± 9.27	111.60 ± 12.56	Time (B)	2, 56	70.787	<0.001 ***	0.717	
					A × B	2, 56	13.507	<0.001 ***	0.325	

Values are presented as means ± standard deviations. EG = aquatic exercise group (participants in the community-based aquatic exercise program); CG = comparison group (participants who did not participate in the aquatic exercise program). Group classification was based on participants’ program participation status and was not assigned by the researchers. The main effects of group (A) and time (B) and the group × time interaction (A × B) were analyzed using a two-way repeated-measures analysis of variance. When the assumption of sphericity was violated, the Greenhouse–Geisser correction was applied. When significant group × time interactions were observed, planned contrast analyses were performed to compare changes across measurement time points (0–8 = baseline to Week 8; 8–16 = Week 8 to Week 16; 0–16 = baseline to Week 16). ** *p* < 0.01, *** *p* < 0.001.

**Table 5:** Longitudinal Changes in Serum Irisin and IL-6 Concentrations in the Aquatic Exercise and Comparison Groups.

Variable	Group	Baseline	Week 8	Week 16	Effect	df	F	*p*	ηp^2^	Planned Contrasts
Irisin (ng/mL)	EG (*n* = 15)	4.15 ± 0.57	4.21 ± 0.36	4.99 ± 0.91	Group (A)	1, 28	0.559	0.462	0.017	EG: 8–16 **, 0–16 ** CG: 0–8 *, 0–16 *
	CG (*n* = 15)	4.75 ± 0.97	4.15 ± 0.63	4.12 ± 0.50	Time (B)	2, 56	1.907	0.160	0.082	
					A × B	2, 56	6.987	0.002 **	0.249	
Interleukin-6 (pg/mL)	EG (*n* = 15)	6.52 ± 3.30	5.07 ± 1.55	4.86 ± 0.88	Group (A)	1, 28	1.190	0.286	0.043	—
	CG (*n* = 15)	6.98 ± 2.78	7.43 ± 6.65	5.05 ± 2.23	Time (B)	1.47, 41.16	2.333	0.125	0.095	
					A × B	1.47, 41.16	0.942	0.372	0.041	

Values are presented as means ± standard deviations. EG = aquatic exercise group (participants in the community-based aquatic exercise program); CG = comparison group (participants who did not participate in the aquatic exercise program). Group classification was based on participants’ program participation status and was not assigned by the researchers. The main effects of group (A) and time (B) and the group × time interaction (A × B) were analyzed using a two-way repeated-measures analysis of variance. When the assumption of sphericity was violated, the Greenhouse–Geisser correction was applied. When significant group × time interactions were observed, planned contrast analyses were performed to compare changes across measurement time points (0–8 = baseline to Week 8; 8–16 = Week 8 to Week 16; 0–16 = baseline to Week 16). * *p* < 0.05, ** *p* < 0.01.

## Data Availability

The data presented in this study are available from the corresponding author upon reasonable request, subject to ethical restrictions related to the inclusion of sensitive participant information.
